# Concrete Strengthening by Introducing Polymer-Based Additives into the Cement Matrix—A Mini Review

**DOI:** 10.3390/ma14206071

**Published:** 2021-10-14

**Authors:** Weronika Kujawa, Ewa Olewnik-Kruszkowska, Jacek Nowaczyk

**Affiliations:** 1 Chair of Physical Chemistry and Physicochemistry of Polymers, Faculty of Chemistry, Nicolaus Copernicus University in Toruń, Gagarin 7 Street, 87-200 Toruń, Poland; janowa@umk.pl; 2 Selena Labs Sp. Z o.o., Pieszycka 1 Street, 58-200 Dzierżoniów, Poland

**Keywords:** cement, additives, superplasticizers, redispersible powders, polymer dispersions, concrete

## Abstract

The modern types of concrete are a mixture of aggregates, cement, water and optional additives and admixtures. In particular, polymer additives seem to be a promising type of component that can significantly change concrete and mortar properties. Currently, the most popular polymer additives include superplasticizers, latexes and redispersible powders. Moreover, in order to improve the properties of concrete-based composite admixtures, which enhance the resistance to cracking, polymer fibres and recycled polymers have been researched. All the types of polymeric materials mentioned above are broadly used in the construction industry. This work summarizes the current knowledge on the different types of popular polymeric additives. Moreover, it describes the correlation between the chemical structure of additives and the macro-behaviour of the obtained concrete.

## 1. Introduction

The cement industry, as it provides building materials for the construction industry, counts among the most significant manufacturers in terms of produce volume. Cement is not a stand-alone building material, although it is the main ingredient of the standard building material called concrete. The latter is the most extensively used building material in the construction industry, has various applications and is mainly used to form structural elements. The widespread use of concrete results from several reasons, such as the abundance of substrate resources, low cost and high compressive strength [1]. Concrete is a mixture of several substances serving different purposes. Typically, concrete contains aggregates such as gravel, sand and/or stones, as well as an adhesive, which is usually cement and water (10–15% vol.). Concrete can be divided into different types. Recently, high-performance concrete is being developed. The high-performance concrete concept started to emerge in the building industry in the 1990s. This new type of concrete is characterized by a low water content (low ratio of water to cement) due to the introduction of plasticizers and secondary binders. Their compressive strength, compared to standard-strength concrete, was raised by no less than 60 MPa after the standard 28-day period [2]. The first report devoted to the super-high compressive strength of concrete, exceeding the 150 MPa value after the standard 28 days, was published by Braunauer et al. [3]. Recently, the rapid development of very tall buildings and large building structures brought about the increased requirements for concrete. The most important features of concrete, translating to high-grade quality, include great compressive strength and exceptional durability. However, the significant weight and brittleness of cement-based composites greatly limits their application in some of the required fields. Due to those restrictions, a new type of concrete was developed and named lightweight concrete (LWC). The typical lightweight concretes are characterized by a density ranging from about 1400 to 2000 kg/m^3^. The lower weight results from the inner voids in their structure. Generally, inner voids can occur when applying cement paste or lightweight aggregates, which have a porous structure. The other approach to obtain inner voids involves the application of coarse aggregate particles [4]. According to previous experience, the compressive strength of lightweight concrete typically decreases with the reduction in density [5]. The correlation of high compressive strength and low density gives the opportunity to potentially use lightweight concrete in the new field of industry, where the weight of material plays a key role. Enhancing the performance and durability of lightweight concrete has been currently the main interest of research in this field.

Currently, the new and most commonly used generation of cement-based composites is ternary and quaternary binder systems, with the addition of different cementitious materials. The ternary binder systems are based on Portland cement (OPC), composed mainly of 3CaO·SiO_2_ (C_3_S), 2CaO·SiO_2_ (C_2_S), 3CaO·Al_2_O_3_ (C_3_A) and 4CaO·Al_2_O_3_·Fe_2_O_2_ (C_4_AF), calcium aluminate cement (CAC), in which the main active constituent is monocalcium aluminate CaO Al_2_O_3_ (CA), and calcium sulphate.. It should be stressed that they can achieve great early strength, rapid setting and shrinkage compensation better than the standard OPC-based materials. In the case of such systems, CAC fulfils a composite superposition effect on the creation of ettringite, whereas gypsum promotes the formation of ettringite [6]. The introduced additives react simultaneously with the hydration progress of the Portland cement clinker and influence the resulting hydrates and concrete microstructure [7,8,9]. The literature review revealed a number of studies involving the possibilities to improve concrete mechanical properties by the addition of polymers to the cementitious matrix. The concrete modified with polymers is called polymer-modified concrete (PMC) or polymer-modified mortar (PMM). Polymers used in PMC have various forms, such as latexes, liquid resins, water-soluble polymers and copolymers, fibres and re-dispersible powders. The type of polymer used depends on the intended application and requirement for concrete properties [10]. Their influence on the structure leads to the improvement of many properties, both fresh mortar and concrete, such as flowability [11], setting time [12], freezing–thawing resistance [13], mechanical properties [14] and anti-penetrability [10,15]. Using polymers to increase the strength and durability of concrete is related to the change in concrete microstructure and shrinkage reducing effect [16,17,18]. The polymers used in this application include film-forming latex polymers, i.e., styrene-butadiene (SBR) copolymer and ethylene-vinyl acetate (EVA) copolymer. The of in the mechanical properties of PMC, such as flexural strength and brittles, have also been proven [19]. Moreover, the cementitious-based composites can also be modified by other additives, such as surfactants, antifoaming systems, viscosity modifiers and stabilizers [10].

Apart from the addition of polymers to the cementitious matrix, scientists are looking for other solutions aimed at eliminating concrete weaknesses, such as its brittleness and its low load capacity. In response to the demand for improved concrete, researchers made an effort to overcome the toughness limitations of plain concrete. In the course of this study, fibres have been examined as an additive incorporated into the cementitious matrix. In recent years, most publications in this field suggest the application of polymer fibres in order to improve the ability to enhance tensile [20,21,22] and flexural performance of resulting concrete [23,24]. The methods of concrete reinforcing by the use of fibres are well known. There are various types of fibres with different chemical structures and Young’s modulus which can improve the dynamic development of the new cement-based materials. Moreover, it was proven that fibres reduce shrinkage cracking of concrete, which affects its durability [25]. Fibre efficiency depends significantly on their chemical nature, amount, dimensions and shape [25,26,27]. Currently, a well-known method of reinforcing cement-based composites involves using polypropylene fibres (PPF). Short- length PPF, characterized by a low modulus, are able to reduce the shrinkage cracks and increase the pre-crack strength, provided they are properly distributed in the cementitious matrix [27]. Additionally, the use of PPF can improve many concrete characteristics, such as fire resistance, abrasive–erosion resistance, tensile strength, bond strength and impact resistance [28]. Various types of polymeric fibres have sparked the interest of scientists in the context of concrete reinforcement. To date, polyamide fibres (PAF), polyethylene terephthalate (PET) fibres, polyethylene (PE) fibres and polyvinyl alcohol (PVA) fibres, as well as hybrid fibres, were tested as an alternative to steel fibres, which are still commonly used in concrete manufacturing technologies. Recently, it was established that synthetic fibres could be replaced with recycled polymer material. This finding is relevant when bearing in mind the critical increase in the quantity of polymer waste, which generates public demand for the reuse of plastic waste. The production of polymer-modified concrete provides an opportunity for the utilization of polymer waste as filler and/or modifier of concrete. Currently, concrete researchers tend to study the potential recycling of wastes originating from industrial post-production scrap, tires, plastic bottles and other materials. Raffoul et al. [29]. showed that tire waste is proportional to tire production, which exceeded 2.9 billion in 2017, with over 300 million tires reaching their shelf life every year in Europe alone. The application of tire waste in the concrete industry has been summarized in the review paper by Siddika et al. [30]. In another paper, Borg et al. [31] have investigated the properties of concrete reinforced with non-biodegradable PET waste. It was shown that the addition of shredded recycled polymer fibres reduces the values of compressive strength between 0.5% and 8.5%. The same study has proven that, due to a 1% addition of 50 mm long fibres, concrete exhibited reduced shrinkage cracking after 28 days.

**Figure 1:**
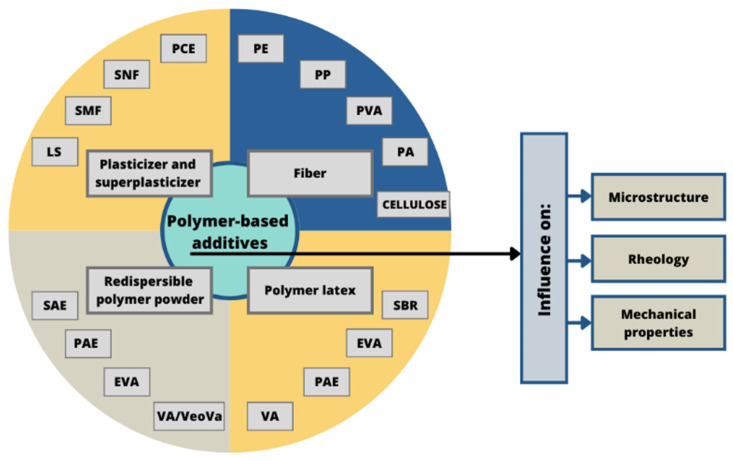
The scheme of the type of additives and the changes they cause in concrete-based composites.

This paper is devoted to the overview of recent developments and research concerning polymer additives used in concrete-based composites. Introducing different polymers in the cementitious matrix can improve the parameters of mortar and concrete. The selection of works discussed in our study focuses mainly on the changes caused in concrete-based materials by the incorporation of various polymer additives (Figure 1).

The review specifically concerns superplasticizers, latexes, redispersible powders, admixtures to enhance crack resistance, fibres and recycled polymers that can play the role of the additives in concrete-based composites. Particular focus has been placed on the relationship between the chemical structure of the described additives and the macro-behaviour of concrete.

## 2. Plasticizers and Superplasticizers

**Figure 2:**
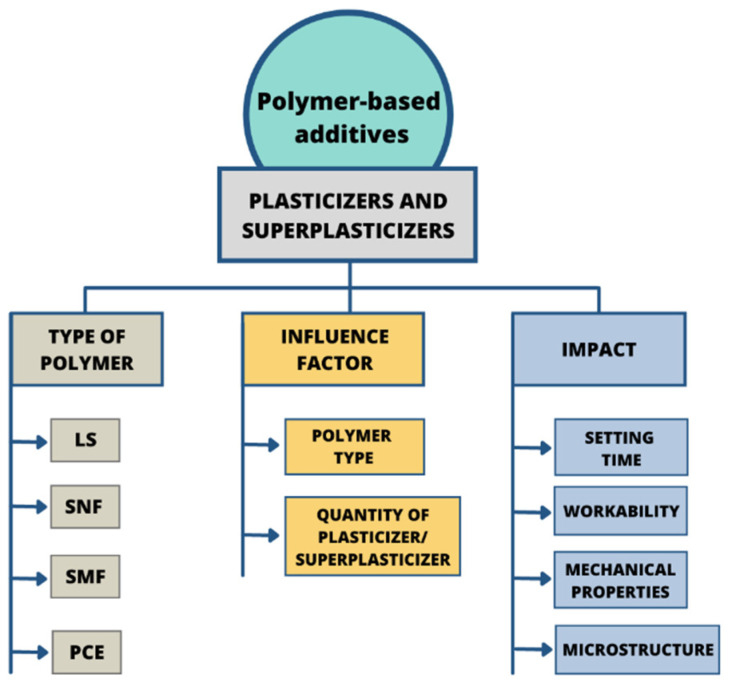
Scheme of plasticizer and superplasticizer types, influence factors and changes caused in concrete-based composites.

The rapid development in the concrete industry in the 1990s was accomplished by the introduction of a new type of chemical admixture referred to as plasticizers. The first generation was based on lignosulphonate compounds, which decreased the water/cement ratio by around 5–10% [32]. It is well known that the reduction of water content in mortar leads to a decrease in the total porosity of concrete [16]. Consequently, decreasing the amount of water in mortar benefits durability and workability, as well as causing shrinkage reduction [33]. Generally, fresh cement paste can be regarded as a suspension dispersion with chemical reactivity. The cement grains, after exposure to water, immediately start to dissolve and hydrate, which leads to the accumulation of both positive and negative charges on the cement surface. Therefore, the flocculation of cement grains occurs as a result of electrostatic interactions between opposite charges on the surfaces of the grains leading to water entrapment [34]. Water present in the mixture could be chemically bonded in hydrates, physically adsorbed on cement grains surfaces, entrapped in the flocculated structures, or remain non-bonded as free water, becoming a dispersion medium. It is well known that the macroscopic properties of liquid–solid dispersion, such as viscosity and flowability, depend on microstructure, which is closely related to liquid–solid interface properties [35]. The addition of plasticizers or superplasticizers to the cementitious matrix induces substantial changes in solid–liquid interface properties (Figure 2) [36,37].

A group of compounds providing further reduction in the water/cement ratio of around 25% is called superplasticizers [38,39,40]. The introduction of these compounds turned out to be a convenient and practical way to improve the properties of concrete. However, the high fluidity they caused could provide adverse effects, such as bleeding and phase separation. In order to eliminate these drawbacks of superplasticizers, the addition of viscosity-enhancing compounds was developed [41,42].

There are a variety of types of superplasticizers differing in chemical structure. They can be classified into groups, such as lignosulphonates, sulfonates, naphthenates, melamine sulfonates and polycarboxylates [43]. The collective list of these compounds is presented in Table 1. In contrast to plasticizers, superplasticizers have a different mechanism of action and, consequently, different effectiveness in reducing the mixing of water. The details of this mechanism are discussed later in this review. Different types of reactive groups present in superplasticizers’ molecules bond the Ca^2+^ cations with different bonding strengths. According to the published data, the strength of these bonds decreases in the following order: phosphate > carboxylate > sulfonate > sulphate > alkoxide and water [44]. Therefore, polycarboxylate containing -COO^−^ groups shows improved effectiveness in dispersing cement particles, compared to, for example, plasticizers containing -SO^3−^ groups. Moreover, attempts were made to research the efficiency of polymers admixtures containing the most effective groups chelating calcium ions, i.e., phosphonates and phosphate groups [36,45,46].

The phosphated comb polymer superplasticizer was synthesized by J. Stecher and J. Plank [45] and its dispersing performance in cement was tested by slump tests in cement paste. They found that the polyphosphate superplasticizers showed improved dispersing performance over PCEs superplasticizers, as well as less retarding on cement. The authors of the paper emphasized that the obtained values result from the high calcium complexing capacity of the phosphate group.

Both plasticizers and superplasticizers are such necessary admixtures in concrete that they are listed in the European standard PN-EN 206, where they are mentioned as a possible solution to achieve the right consistency by reducing the amount of water, or reducing the cement content, or to modify consistency without changing the amount of water and cement [52].

In order to reduce the water volume and achieve higher solid content while maintaining proper consistency, superplasticizers, called high-range water reducers, are often used in the formulation of concrete. Scientists and engineers have determined the basic properties of superplasticizers and confirmed that their use allows manufacturers to improving the workability of fresh concrete [53], chloride binding [54] and durability of cementitious materials [16]. However, the application of such compounds has brought many new problems resulting from the poor compatibility of superplasticizers with the multi-component system of concrete, including cement, fine aggregate and additional materials. The most significant problems include bleeding, segregation, low initial slump, flash set and set retardation [55]. Therefore, scientists have started to thoroughly investigate the interactions between superplasticizers and particles in the cementitious matrix.

N. Roussel et al. [56] have indicated that the rheological properties of fresh concrete are determined by the interactions among the particles in the cementitious matrix. While the cement hydration process is ongoing, new rigid phases are created, such as anhydrous phase at an early stage, ettringite, calcium silicate hydrate (CSH) and gypsum. It leads to an increase in yield stress, thixotropy and hardening of the cementitious material, significantly reducing the workability [36,55]. The application of superplasticizer serves to keep distance among the particles through the distribution of polymer molecules adsorbed onto the particles’ surfaces, decreasing the strength of the interactions. The improved workability results from releasing a large quantity of water, which reduces the effective solid volume fraction during the deflocculation process [34,55].

Many studies have shown that the fluidity of fresh cement paste depends on the amount of superplasticizer adsorbed by a square meter of solid particles [57,58,59]. The adsorption of superplasticizer on the solid surface depends on the polymer’s chemical structure, a solid interface and pore solution containing different dissolved ions [36]. It should be stressed that the processes of superplasticizer adsorption and cement hydration occur at a certain point of concrete formation [60]. At the early stage, during the first period of cement hydration, new phases develop, leading to morphology changes in the cementitious matrix. Superplasticizers display different adsorption affinity toward the surface of various hydration products and mineral phases, affecting the hydration process, including ettringite formation. Therefore, the hydration rate, mortar morphology and surface properties of the hydration products, as well as their size and amount, greatly influence the concrete workability [61,62,63].

In the case of Portland cement hydration, the main composition of solid surfaces is a result of the hydration of calcium silicate and aluminate phases. Among them, certain interfaces can be specified separating the ettringite, gypsum, CSH and anhydrous phases. According to previous studies, ettringite shows the highest adsorption capacity for superplasticizers. The CSH phase adsorbs superplasticizers at least 3–10 times less than ettringite. Consequently, ettringite is considered to be the crucial phase to understand the fresh concrete rheology [36]. Marchon [64], in his work, has indicated that the ettringite surface is entirely covered by the polycarboxylate superplasticizer (PCE). Liu et al. [55] have provided an elucidatory description of the interaction between polycarboxylate polymer molecules and the hydration products of OPC. The surface coverage of cement hydration products by PCE together with the size distribution of the cement particles affects the workability of the obtained material. Polycarboxylate superplasticizer strongly adsorbs onto positively charged cement particles. However, the adsorption process could be weakened through the screening effect of counterions.

The other recently analysed super plasticizer is Welan gum [39]. In the work by Khayat et al. [65], it was established that Welan gum, at low shear rates, is able to increase yield stress, enhance rebuilt-up kinetics at rest and increase the viscosity of cement paste. It is noteworthy that Portland cement is not the only type of cement used in practice; there are also other types that have different mineral composition. H. Tian et al. [66] have investigated the effects of polycarboxylate superplasticizers on sulfoaluminate cement (SAC)systems. SAC contains the calcium sulfoaluminate fraction and various calcium sulphates, such as CaSO_4_, CaSO_4_·2H_2_O and CaSO_4_·0,5H_2_O, responsible for faster hydration of SAC as compared to OPC. In the article by H. Tian et al. [66], the effects of polycarboxylate superplasticizers, obtained via the radical copolymerization of acrylic acid (AA) and a-methallyl-x-hydroxy poly(ethylene glycol) with a monomer ratio of 4, an ordinary Portland cement, have been investigated in the SAC system. The study confirms the differences in compatibility between the selected superplasticizer and the cement used.

**Figure 3:**
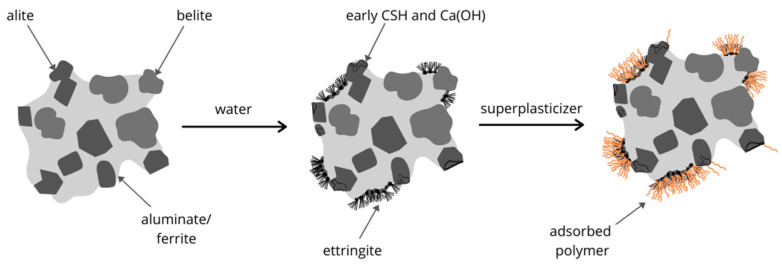
The scheme of a hydrating cement grain with uneven superplasticizer polymer distribution on its surface (adapted from [67]).

The adsorption of the superplasticizer on the solid surface depends on the chemical structure of the polymer. The added superplasticizer is adsorbed onto the surface as a consequence of electrostatic or specific interaction with the interface (Figure 3).

It is a well-known fact that the type of functional group of superplasticizer is characterised by a different adsorption affinity toward a given surface. This phenomenon was discussed by J. Stecher and J. Plank [45]. The authors synthesized phosphate comb superplasticizers based on methacrylate ester and compared their properties with their carboxylate counterparts. They found that polyphosphate comb polymers outperform the polycarboxylate ones in terms of their dispersing capacity in cement paste, attach more readily to the cement surface and impede cement hydration to a less significant degree [45]. Apart from the type of functional group of superplasticizer, other parameters of polymers that have a significant influence on the adsorption process should be mentioned. These parameters include the number and the density of the adsorbing groups and the length of the side-chain, as well as its grafting density [55,68]. Many studies indicate that the dispersing efficiency of the PCE superplasticizer depends on either the dosage and quantity of PCE adsorbed on the surface of the cement particles or the charge density and activity of the long side chain [69,70,71]. Moreover, it was established that the efficiency of the additive used in the procedure significantly depends on the adsorption of superplasticizer onto the cement particles [40].

It has also been noted that superplasticizers impact the setting time of fresh concrete and the mechanical properties of concrete. Polycarboxylate superplasticizers display the property of slowing down ettringite formation but increase its total surface area [62]. It was found that PCE superplasticizers facilitate the formation of nano-sized ettringite, which is the primary source of incompatibility between cement and additives [61]. Moreover, PCE adsorbs onto the reactive sites of 3CaO·SiO_2_ (C3S), inhibiting its dissolution and delaying the hydration process [64]. Shen et al. indicated that the addition of PCE to SAC could delay the setting time of cement-based substances [72]. Furthermore, a superplasticizer can positively affect the mechanical properties of concrete. The reason for an increase in the compressive strengths of concrete can be explained by a microstructural improvement, especially due to the reduction in the water/cement ratio. Several studies have investigated the influence of superplasticizers on the mechanical and rheological properties of mortar and concrete. Researchers have established that the effect of superplasticizers on the performance of hardened concrete depends on the type and dosage of the superplasticizer used as well as the binder [43,68,73]. M. Benaicha et al. [70] have presented the correlation between the rheology and the strength of self-compacting concrete (SCC). In that case, the compressive strength decreased with the increase in the amount of superplasticizer.

**Figure 4:**
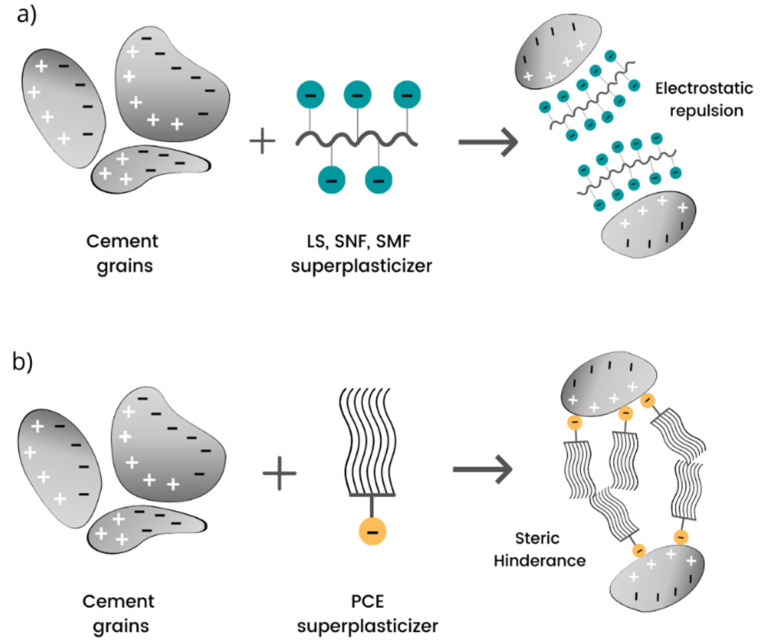
Schema: (**a**) electrostatic repulsion effect of cement; (**b**) steric hindrance mechanism, (adapted from [51]).

It is well known that superplasticizers act as dispersants in colloidal particle suspensions that prevent undesired agglomeration and reduce overall viscosity. In order to allow the cement paste flow, the yield stress associated with the network of rigid particles has to be exceeded. The yield stress of cement paste is connected with colloidal and contact interactions among the particles and it depends on the nature of solid particles and their volume fraction [55]. Superplasticizers may have a different mechanism of interaction with cement particles, directly related to their chemical structure. In general, one can distinguish two mechanisms of their interaction, corresponding to electrochemical and steric hindrance forces [74,75]. In Figure 4, two different mechanisms of action of superplasticizers in the cementitious matrix are presented.

The mechanism based on electrochemical forces was first developed to explain the properties of plasticizers such as lignosulphonate. Lignosulphonate compounds have a bipolar structure and display properties typical of polyelectrolytes. The mechanism of their interaction is based on the physical repulsion of negatively charged cement particles, leading to the disintegration of cement lumps into smaller particles, which decreases the surface tension on the surface of the grains wetted by mixing water. Consequently, fine cement grains move more quickly [47]. Electrostatic repulsion results from an increase in the zeta potential, which depends on the presence of the negative charges in the cementitious matrix [76,77]. Both naphthalene and melamine have a similar working mechanism to the one observed in lignosulphonate plasticizers, providing an electrical dispersing effect [74].

In contrast to linear polycondensates, which disperse cement particles via electrostatic repulsion, PCE molecules, having comb-shaped structures, achieve the dispersing effect mainly via steric hindrance [46,65]. In general, polycarboxylates-based superplasticizers, including polyacrylates, acrylic esters and sulfonated polystyrene, consist of negatively charged backbone carboxylic groups and lateral grafted chains. The latter ones are composed of ethylene oxide units (EOUs) [78]. The steric hindrance effect results from the oriented adsorption of the superplasticizer molecules on positively charged cement surfaces and leads to the weakening of the attraction between the cement particles. The negatively charged carboxylate anions at the polymer backbone adsorb on the positively charged surfaces of the cement particles. At the same time, grafted side chains hinder the aggregation of cement particles, introducing a steric repulsion and a fluidizing effect. Once the adsorption of polycarboxylate superplasticizer occurs, the particles’ zeta potential, from positive, becomes negative, or zero [75]. As a result of attaching superplasticizers to the cement particles, they cannot approach each other and the attraction forces among the cement particles are weakened. Numerous studies have shown that the fluidity of mortar depends on the amount of superplasticizer adsorbed on the particles’ surfaces [57,58].

A different approach to superplasticizers and their interactions with cement has been presented in the works by Flatt and Hust [79] and Flatt et al. [80]. According to their theory, the introduced superplasticizer is divided into three parts. They have established that the first part of the superplasticizer is utilized during chemical reactions. The second part is adsorbed onto the cement surface, while the last part constitutes the superplasticizer, which forms a saturated system after the introduction of an adequate volume of the additive. Moreover, according to the work of Qian et al. [34], it should be stressed that the increase in the PCE superplasticizer concentration leads to the increase in both the adsorbed part and the remaining part of the additive.

## 3. Redispersible Powders and Polymer Dispersions

The rapid development of the construction industry induced the pursuit to improve the basic properties of concrete and to overcome its limitations, such as brittleness, low durability and insufficient strain capacity, through modifications of the microstructure of hydrated cement. In recent years, the innovations in building construction have progressed considerably and the research on high-performance cement-based materials has been furthered to cope with the requirements of the industry. One of the possibilities to improve concrete performance, including strength and durability, is to introduce polymers into the cement matrix. Cement–polymer composites are created by substituting all or a part of the cement hydrate binder with polymers. Polymer-modified concrete was first introduced in the 1990s and is commonly being used as one of the typical construction materials [10].

**Figure 5:**
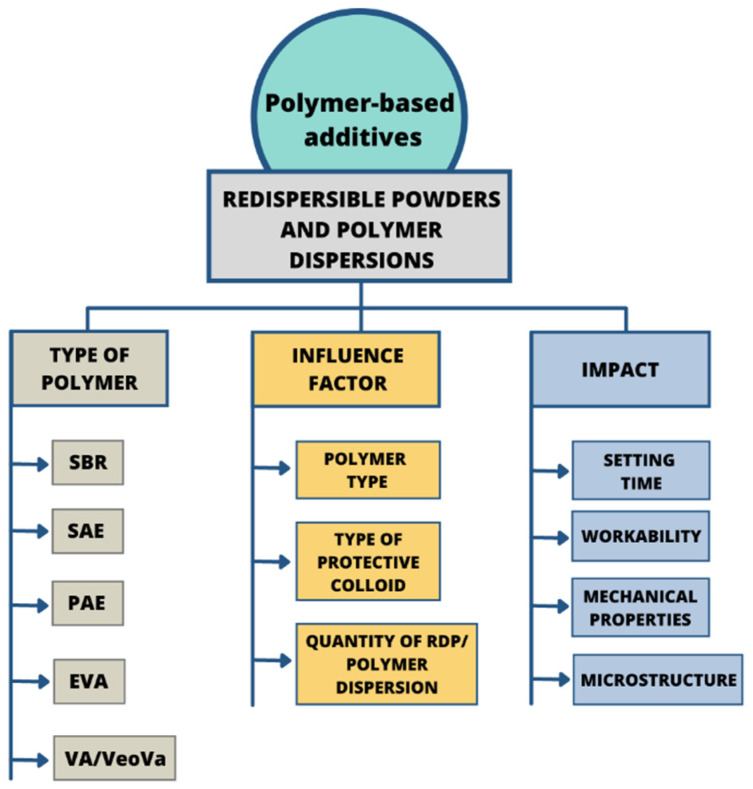
Schematic representation of redispersible powders and polymer dispersion type, influence factors and changes caused in concrete-based composites.

Nowadays, various types and forms of polymers are used as chemical admixtures (Figure 5). Among those widely used additives are polymers with a different chemical structure, such as lignosulfonates, polyvinyl acetate, ethylene-vinyl acetate, styrene-butadiene copolymers, styrene-acrylic and polyacrylic ester [81,82], which are presented in Table 2. Many studies have been reported about these materials [12,83,84,85,86]. Currently, polymers are applied in cement in various forms, such as latexes, liquid resins, redispersible powders and water-soluble homo- or copolymers [81]. Considering the fact that the type of the polymer used in the manufacturing process influences the properties of the resulting composite, the selection of a polymer type and form depends on the intended use of concrete and is associated with its desired properties, such as strength, chemical resistance and durability [87].

Initially, polymer-modified mortar and concrete were produced by the addition of a polymer dispersion in latex or emulsion form to the plain cement-based composition during the mixing process. The main advantages of polymer latexes are their ability to create flexible polymer films after dehydration, as well as providing proper adhesion and cohesion in cementitious materials [12]. As mentioned before, water-based polymer systems are used in order to improve the properties of ordinary concrete and contribute to increasing mechanical strength [10], improving workability [10,86] and durability [13], reducing water absorption [88] and causing a decrease in total porosity [89]. The possibility of re-emulsification in humid alkaline conditions is one of the limitations of these polymers [10]. Due to their superior properties, cement–polymer composites are used in various applications, such as repair mortars, waterproofing membranes, self-levelling compounds and tile adhesives.

Redispersible polymer powders (RDP) are a modern type of substances produced by spray-drying polymer dispersions and often used for the same purpose as polymer latexes. They are spray-dried to receive polymer powders [81]. It is important to emphasize that spray-drying auxiliaries strongly influence the properties of the RDP-modified mortar. It was observed that polyvinyl alcohol (PVA), which is an example of a colloidal stabilizer in the production of carboxylated styrene-butadiene latex, tends to screen the negative charges of polymeric carboxylate groups, which are to react with calcium ions. As a result, the process of forming a polymer film does not occur properly and the conversion from stage II to stage III is accelerated, as shown in Figure 3 and described in Section 3.1 [90,91].

The properties of cement–polymer concrete and mortars obtained using RDP powders are comparable to those formed in the process of polymer dispersion [93]. The major difference between these two types of concrete is the presence of the spray-dried auxiliaries in the first of the mentioned concrete types, which affects the composite properties. The spray-drying compounds adsorbed on the polymer surface need to dissolve or disperse from the polymer surface to allow coalescence and film formation. This requirement makes the polymer latexes less viable. Consequently, in many cases, they are replaced by an admixture of redispersible polymer powders because of their more straightforward application in concrete production.

### 3.1. Mechanism of the Polymer Film Formation in a Cementitious Matrix

The incorporation of polymer into the cementitious matrix changes the microstructure of concrete [94]. The impact of polymer addition on the cement hydration process was thoroughly investigated in recent years. The nature of the interaction between polymer and cement particles is a subject of ongoing debate among scientists. Many studies explain the physical interactions between the binders and the polymeric film formed inside the cementitious matrix and their contribution to hardened mortar and concrete properties. Researchers have also reported both chemical and physical interactions between cement and polymers, theorizing the formation of new complex structures and changes in the morphology of cementitious materials, such as the composition and quality of hydrated phases [14].

**Figure 6:**
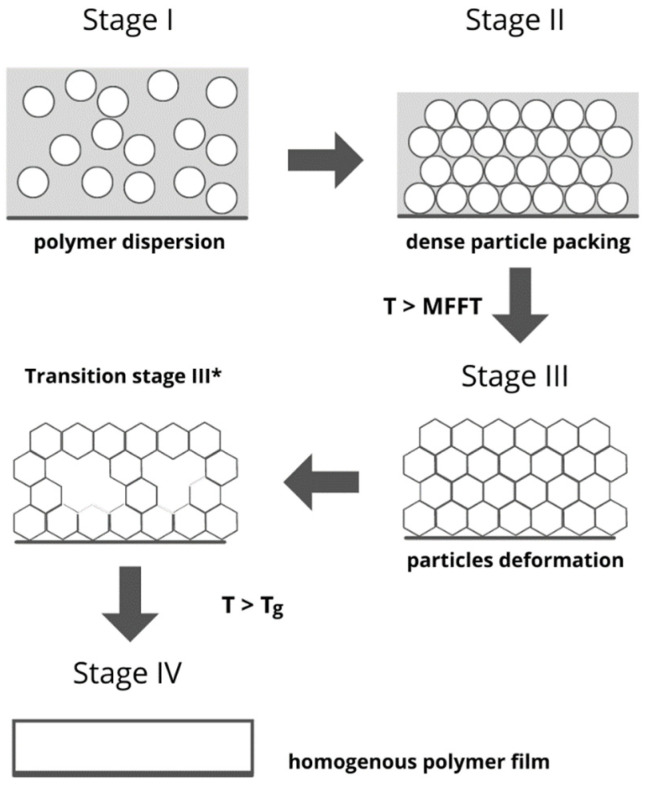
Schematic representation of polymer film formation from aqueous dispersion, stages from I to IV (based on literature [95]).

Polymer film formation is a multistep phenomenon in which four stages have been distinguished (Figure 6).

In the first stage, the polymer particles are dispersed in water (solvent). The evaporation of water leads to the agglomeration of polymer particles and the formation of the second stage, consisting of a close-packed array with entrapped water in the interstices. The next stage is a consequence of water expulsion from interstices by hydration and evaporation processes and is characterized by a dense array of hexagonal, deformed polymer particles. Some researchers point to an additional, intermediary stage (Stage III*) occurring between stage III and stage IV, formed of a randomly packed array of deformed particles surrounded by water-filled interspaces. Finally, stage IV is formed as a result of the coalescence of polymer particles into a homogeneous polymer film. The transition from stage III to IV is possible only if the ambient temperature is above the glass transition temperature (T_g_) of the polymer [95,96].

### 3.2. Mechanical Properties of Cement–Polymer-Based Materials

Redispersible polymer powders mixed with water produce homogenous dispersion with characteristics similar to the original polymer dispersed in water. The polymer film formation is a result of the coalescence of individual latex particles after their dehydration. These chemicals are designed to be dispersed only once. For this reason, if hardened concrete becomes wet again, they stay unchanged. In fact, the formed films display increased cohesion during the fresh state and adhesion in the hardened state [97].

Polymers can improve the basic parameters of concrete such as mechanical properties, the flowability of fresh mortar, anti-permeability and freezing-thawing resistance, as well as anti-corrosion. Research results show that the addition of polymers into the cementitious matrix significantly alters its microstructure and the strength of physical and chemical interactions in the cementitious phase [15].

It is quite evident that different polymers have a different impact on mortar and concrete (Table 3). Many works have been published regarding the attributes of polymer-modified cement materials. According to some reports, styrene-butadiene rubber (SBR) latex improves the flexural and tensile strength, carbonation resistance and waterproofing properties, as well as anti-shrinkage, of the mortar. Ethylene-vinyl acetate copolymer latex, which is the most extensively used polymer in concrete technology, results in an output similar to SBR, additionally increasing the flexural and tensile bond strength and concrete durability. Styrene-acrylic ester (SAE) copolymer latex increases the durability but reduces the elastic modulus of cementitious materials [6,12,15,89,92,98]. Similar results were obtained during the study of the effects of applying redispersible powders in cementitious materials. Many researchers have confirmed that the application of RDPs improves the mechanical strength of mortar and concrete, e.g., the compressive strength and flexural strength, which shows a gradual increase depending on the content of the RDPs used in the process. Furthermore, it is generally agreed that the polymer powders’ admixtures affect several properties of concrete, such as freeze-thaw resistance, water permeability, elasticity modulus and corrosion resistance [18,98,99].

Water permeability and curing conditions are among the most important factors that can lead to the deterioration of mechanical properties and, subsequently, affect the service life of concrete. Young-Kug Jo [92] has examined the microstructure of polymer-modified concrete after curing in different conditions and the effect of curing conditions on the adhesion in the tension of concrete. In the study, three polymers—ethylene-vinyl acetate (EVA), styrene-butadiene rubber and styrene-vinyl acrylic ester—were tested in standard, dry, water and high temperature (70 °C) curing conditions. The adhesion in the tensile adhesion of concrete, modified by means of polymers, depends on both the type of polymer and the curing conditions. The maximum adhesion in the tension of concrete was achieved using EVA in standard curing conditions. Dry curing conditions provided the proper drying time for the polymer film-forming. However, in humid conditions, the polymer film was not uniformly dispersed in the cementitious matrix and did not completely form a three-dimensional lattice structure. As a result, the adhesion in tension after water curing was lower [92].

S. Gwon et al. [18], in their study, examined the influence of an acrylic redispersible polymer powder addition on the microstructure development and mechanical properties of ultra-rapid hardening concrete. The main aspects of the investigation were compressive strengths, rheology, hydration phase evolution, porosity and morphological transition, as well as setting time. The obtained results have shown that the addition of a polymer delayed the setting time but significantly reinforced the microstructure of the ultra-rapid hardening cement systems. The researchers specified the optimum polymer-to-cement ratio as about 10% of polymer-based content in the test results [18].

The increase in compressive tension and direct-shear bond strengths resulting from the application of EVA or acrylate polymers to cement blends was confirmed by Medeiros et al. [14]. Silva and Monteiro [100] examined the influence of EVA powder and cellulose ethers on the hydration of Portland cement phases, especially C_3_S and C_3_A. High-resolution microscope imagery revealed the effects of polymer addition on nucleation and growth of hydrates. The EVA copolymer particles tend to agglomerate around C_3_S grains during the hydration process and seem to act as nucleation sites for the CSH phase. However, the introduction of EVA into the cementitious matrix also has a significant drawback, preventing ettringite formation in the first hours of cement hydration [100]. Other studies involving vinyl acetate and versatate copolymer (VA/VeoVa) have shown that this compound possesses improved resistance to alkaline hydrolysis compared to other copolymers containing vinyl acetate groups [101]. Additionally, VA/VeoVa powder has good water-reduction effects and shows water-retention in the cement mortar. Consequently, the toughness of concrete is significantly improved and the shrinkage rate is reduced. Furthermore, the presence of the VA/VeoVa powder facilitates air entrapment, leading to increased total air content in fresh mortar. In the work of Wang et al., it has been shown that VA/VeoVa polymer powder depresses the compressive strength of concrete [15].

### 3.3. Aging of RDP-Modified Cementitious Blends

The presence of water leads to a partial prehydration of the cement surface and the polymer film formation. PVA present on the RDP particles’ surface, which is a colloidal stabilizer in the spray-drying process, might dissolve in humid conditions, allowing polymer powder to coalesce into a film, partially protecting the cement particles from aging. As a result, the mechanical properties, such as compressive and flexural concrete strengths, with RDP powder achieve higher values than concrete without RDPs after conditioning in humid air.

There is little information in the literature pertaining to the long-term performance and durability of cement–polymer concrete, especially with redispersible powder polymers. J. Schulze and O. Killermann [102] examined and described a long-term performance of three different RDPs admixtures, i.e., vinyl acetate-ethylene, styrene-acrylic and ethylene-vinyl chloride-vinyl laurate. They have established that the morphology of the polymers in the cementitious matrix does not change over the 10-year long storage, neither in dry nor humid conditions. The polymer particles were distributed in the matrix and formed secondary reinforcement in the pores and flaws of cementitious blends. Cement is an inorganic binder responsible for compressive strength, while the redispersible powder, which is an organic binder, influences the internal tensile strength and adhesion bond strength at interfaces. Both cement and redispersible powder act in synergy, improving the properties of mortar and concrete [19,102].

## 4. Fibres

**Figure 7:**
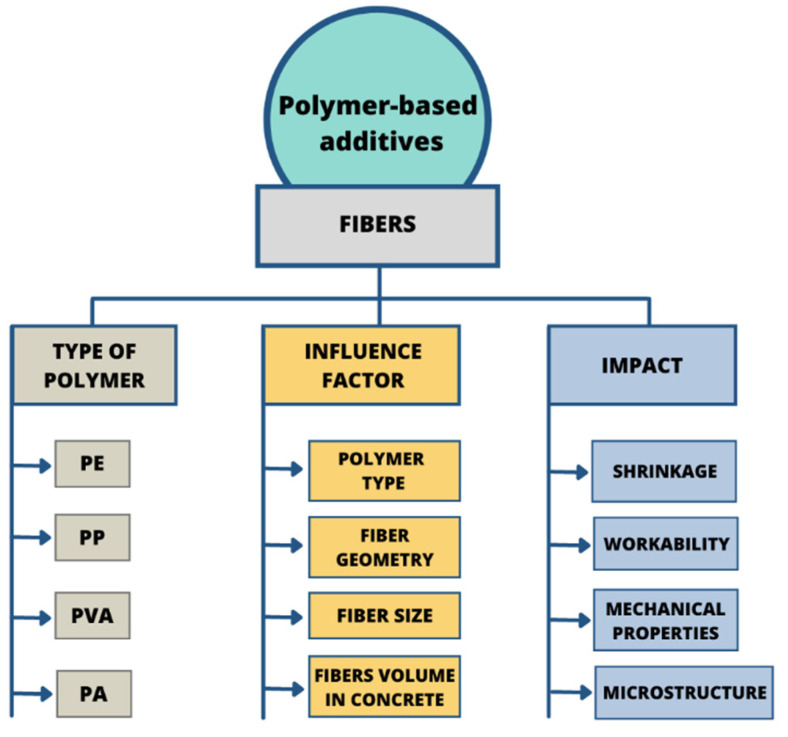
Influence of polymeric fibres on the properties of concrete-based composites.

The review of the literature revealed a number of studies involving the application of fibres in cementitious composites. These materials are called fibre-reinforced concrete. An addition of fibres to a cement-based blend improves the mechanical properties, toughness, ductility and post-cracking resistance of mortar and concrete. During the last decades, a variety of different types of fibres have been examined in cement-based materials. Steel fibres, glass fibre, natural and polymer fibres are the fibres predominantly used in order to reinforce concrete. The traditional use of steel fibres has many advantages, e.g., it greatly enhances the tensile strength and flexural strength of cementitious materials. This phenomenon results from the steel fibres’ capacity to absorb energy and the capability to control cracks. Moreover, due to their high electric and heat conductivity, steel fibre-reinforced concrete can be applied in special conditions. However, the corrosion of steel is a significant disadvantage of these materials and can potentially compromise the durability of the resulting structure. Glass fibres and natural fibres, such as wood, coconut, palm and vegetable fibres have a good strengthening effect, but their application has significant drawbacks. Glass fibres have poor alkali resistance, while natural fibres have poor durability. The usage of randomly distributed polymer-based fibres in the cementitious matrix has received broad attention because of significant effectiveness in improving the basic characteristics of concrete (Figure 7).

Immunity to corrosion and alkaline reactions, salts, chlorine and microorganisms count among the most beneficial results achieved by applying polymer-type fibres.

**Figure 8:**
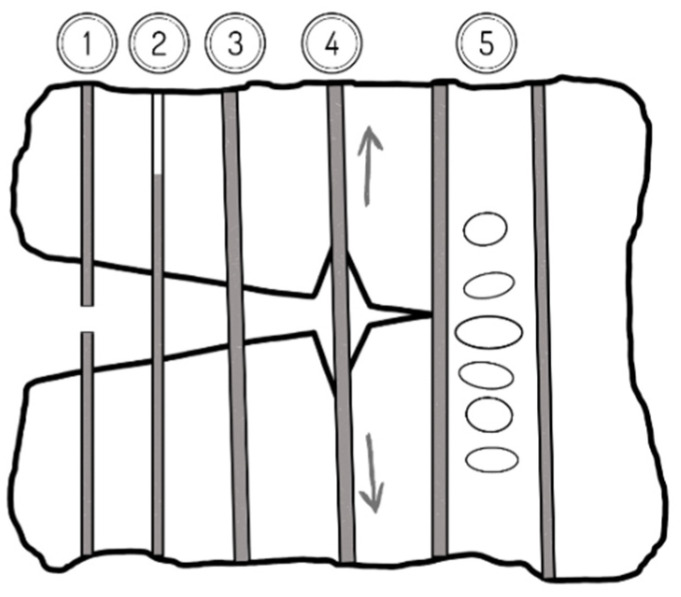
The schematic of energy absorption between concrete and fibres (based on literature [103]). (1) Fibre failure; (2) fibre pull-out; (3) fibre bridging; (4) concrete matrix/fibre debonding; (5) concrete matrix cracking.

**Figure 9:**
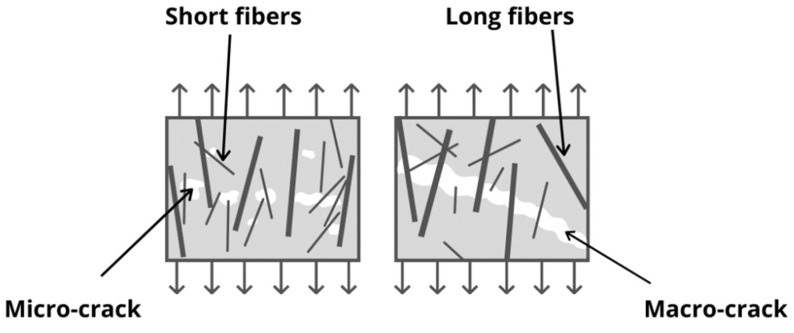
The blocking of crack propagation in fibre-reinforced concrete (based on literature 107).

R.F. Zollo [103] has published a schematic mechanism of crack arrest for fibre-reinforced concrete (Figure 8). The schematic diagram shows the potential of the fibres to absorb energy and control crack propagation. The diagram depicts the fibre rapture (1) and its pull-out (2), bridging by tension through the fibre (3) and debonding of fibre from the matrix, which can effectively dissipate energy to prevent crack growth. The presence of fibre in the matrix (5) helps restrain the cracking area and in consequence, smaller cracks are distributed in the adjacent space of the cementitious matrix, as is shown in Figure 8. The reinforcing effect observed in concrete is not the result of the individual fibres, but a cumulative effect of all fibres. In conventional concrete, micro-cracks are presented even before the concrete is loaded. Their occurrence is caused by the drying shrinkage, leading to volume contraction. Some researchers have reported a reduction in drying and plastic shrinkage cracks because of the use of fibres [25,104]. Generally, the micro plastic fibres, whose length is 5–30 mm and whose diameter ranges from 5 to 100 µm, can effectively reduce plastic shrinkage cracking. The blocking of crack propagation in concrete by micro and macro-fibres is presented in Figure 9. The macro-plastic fibres ranging from 30 to 60 mm in length are used to control shrinkage, mainly drying shrinkage. The formation of plastic shrinkage cracks can be linked to moisture loss after casting. If the moisture evaporation rate is greater than 0.5 kg/m^2^ per hour, it can bring about internal strain induced by the rising negative capillary pressure inside the matrix. Plastic shrinkage occurs in the initial stage when the strength of concrete has not developed yet [105]. Kim et al. [25] tested the influence of fibre geometry and volume fraction on the rate of moisture loss and plastic shrinkage cracking. They have found that the volume of macro-plastic fibres and their geometry do not affect the total moisture loss, while plastic shrinkage was reduced when the fibre fraction equalled 0.25%.

The literature indicates that the most commonly used synthetic fibres in concrete are polypropylene (PP) fibres, polyamide (PA) fibres, polyethylene (PE) fibres and polyvinyl alcohol (PVA) fibres. In Table 4, the basic properties of each of the mentioned synthetic polymer fibres are summarized and compared with steel and cellulose fibres. All of them are characterized by a low density, which causes a high volume of fibre content in the cementitious matrix compared to the relative mass of the fibres.

In the last decades, researchers investigated the influence of synthetic fibres on the rheology and mechanical properties of cement-based materials. Scientists have studied the effect of various types of fibres, differing in chemical structure, volume in the cementitious matrix, size (macro- and micro-) and geometry [25,108]. Moreover, the impact of single and hybrid (type of length and size) polymer fibres on concrete has been examined [27,110].

### 4.1. Rheology Behaviour and Mechanical Properties of Cementitious Materials Containing Fibres

The addition of fibres to the cementitious matrix influences both the fresh and the hardened state of concrete. It is well known that polymer fibres reduce the workability of fresh mortar [106]. This phenomenon results from the formation of a network structure in a concrete matrix which inhibits the flow of the blend. Furthermore, a high volume and surface area of fibres can lead to adsorption of water, hence increasing the viscosity of mortar [105,111]. However, Ramezanianpour et al. [28] considered that the reduction in workability of mortar with a higher quantity of fibre inside the cementitious matrix is due to the presence of air entrapped in the inner pores. M. Tabatabaeian et al. [106] have investigated the rheological properties of fresh concrete reinforced by fibres. Their study shows that the addition of steel fibres causes a slight decrease in slump flow, while polypropylene fibres significantly reduce the slump flow. In the study by Yap et al. [112], the workability of the mortar has been shown to depend on the micro-fibre geometry. The fibrillated polypropylene fibres have a lower effective surface area, which results in better workability in fresh concrete compared to multifilament fibres [113]. It was reported that hydrophobic polyolefin fibres, such as polypropylene and polyethylene, have a similar influence on concrete properties, including the rheology of the fresh blend. Researchers have observed that PE fibres decrease the slump flow, to an extent similar to the one observed in the case of the incorporation of PP fibres [112,114,115]. The presence of PVA fibres leads to a reduction in workability proportionate to an increase in fibre content. The effect PVA fibres have on the reduction in flow is more significant than that of PE and PP fibres, which is explained by the hydrophilic nature of PVA [114].

As mentioned earlier, the fibres’ effect on the mechanical strength of concrete depends on their size and chemical structure and on the quantity of fibres in the cementitious matrix (Table 5). The research study by Yin et al. [105] indicates that macro-plastic fibres in cementitious materials have no significant impact on compressive strength. That is consistent with what was reported by Behfarnia and Beharvian [116]. However, the research study conducted by Felekoğlu et al. [117] showed that the addition of PP macro-fibres increased the compressive strength of foamed concrete.

Furthermore, the improvement of compressive strength of self-compacting concrete due to the incorporation of PP macro-fibres has been confirmed by Gencel et al. [118]. Ramezanianpour et al. [28] investigated the effect of PP fibres on the mechanical characteristics of concrete intended to be used in sleepers. They reported that the addition of an increased fibre amount of PP fibres to concrete sleepers gradually decreased the compressive strength. Moreover, the influence of PP fibres on the mechanical properties and durability of high-strength SCC in comparison to steel fibres was examined by M.Tabatabaeian et al. [106]. The tests showed that incorporation of PP fibres led to a depletion of compressive strength in comparison to the control mix. Additionally, a replacement of steel fibres with PP fibres in hybrid mixes caused a reduction in the compressive strength in the case of all the hybrid samples.

In recent years, researchers have focused on hybrid fibres because of their superior effects on composite performance compared to mono fibre blends. Chen and Liu [110] tested single and hybrid types of fibres in high-strength lightweight concrete. The single PP fibres caused a decrease in compressive strength with respect to the reference samples, while hybrid fibres, including PP fibres, showed the smallest impact on concrete properties. Hsie et al. [119] examined the mechanical properties of concrete reinforced with polypropylene hybrid fibres. The results revealed that the mixed PP macro- and micro-fibres added to the cementitious matrix increased the compressive and flexural strength as compared to mono fibres. Yun has studied the mechanical properties of concrete reinforced with the PVA/ultra-high molecular weight PE hybrid fibres. The test results showed that a higher amount of PVA fibres in the hybrid samples improved the compressive performance in comparison with the samples containing an increased amount of PE fibres [119]. Guler [108] has investigated the use of PA fibres in hybrid form in cement-based composites. According to the presented results, the addition of both PA macro- and micro-fibres in either a single or hybrid state did not cause a noticeable rise in the compressive strength of reinforced concrete, whereas the flexural strength increased significantly.

Cao et al. [109] have created a new multiscale hybrid fibre system consisting of CaCO_3_ whiskers, PVA and steel fibres. CaCO_3_ whiskers are a novel type of micro-fibres that are able to significantly improve the mechanical properties of cementitious materials. The previous tests proved that cement-based composites reinforced with multiscale hybrid fibres could improve the flexural strength, energy absorption capacity and reduce the plastic shrinkage of concrete.

### 4.2. Microstructure of Synthetic Fibre-Reinforced Cement-Based Materials

The microstructural analysis is an appropriate method that allows us to understand the physical and mechanical properties of fibre-reinforced cementitious materials. Therefore, scientists pay considerable attention to the microstructural characterization of concrete. The literature review has revealed many articles focused on the study of microstructures present in fibre-reinforced concrete by means of several techniques, such as scanning electron microscopy (SEM) [28,120,121,122], energy dispersive spectroscopy (EDS) [123], X-ray diffraction analysis (XRD) [26,120,121,124,125], infrared absorption spectroscopy (IR) [123], Fourier transform infrared spectroscopy (FTIR) [120,123] and thermogravimetry analyses (TGA) [124].

**Figure 10:**
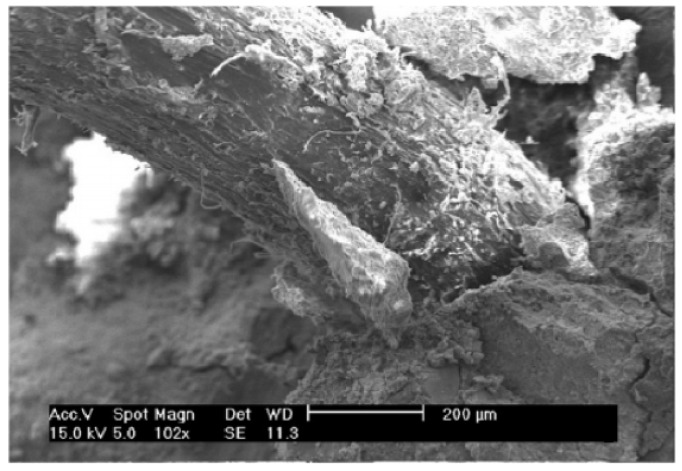
Poor bonding and entrapped air voids around a PE fibre in concrete matrix [131].

**Figure 11:**
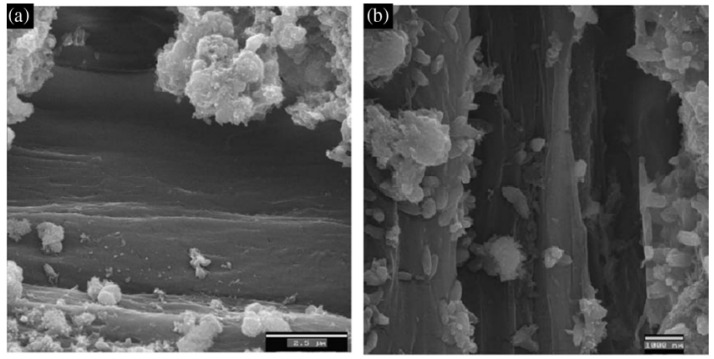
The surface of polypropylene fibres: before alkaline treatment (**a**) and after alkaline treatment (**b**) [128].

Generally, the hydrophobic nature of polymer plastic fibres causes their poor bonding in hydrophilic cementitious materials. The SEM analysis shows the presence of entrapped air voids around polyolefine fibres (Figure 10). In order to mitigate this problem, some researchers modified the surface of fibres using chemical solutions [126,127,128] and plasma treatment [129]. Lopez-Buendia et al. [128] have discussed surface modification of polypropylene fibres by means of alkaline treatment (Figure 11) and cement crystal growth on the surface of the modified fibres, which resulted in better fibre–cement adhesion. The literature review shows that surface treatment causes improvement of mechanical parameters of hardened concrete, such as flexural strength [127,129], crack strength [126] and toughness [126,129].

**Figure 12:**
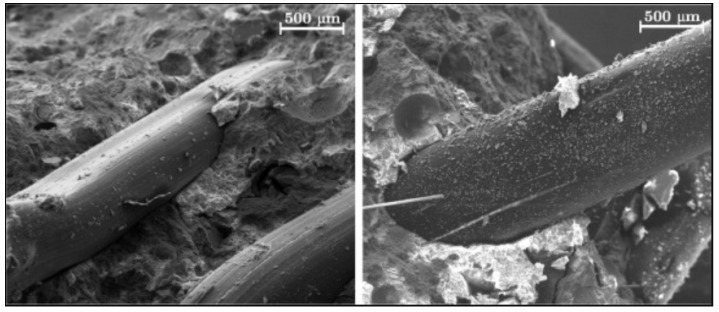
The surface of samples after bending test: non-modified PP fibres (**left**) and silica-coated PP fibres (**right**) [130].

Another experimentally tested method of surface modification of fibres is thin layer coating. Hernandez-Cruz et al. [129] have investigated the chemical interactions between PP fibres covered by ethylene acrylic acid copolymer (EAA) and cementitious matrix. They have found that, in the cementitious paste comprising the EAA-covered fibres, bonding is improved because of the hydrophilic carboxyl groups presented in EAA that interact with the Ca^2+^ and Na^+^ cations from the cement paste. The improved bonding enhances the post-cracking behaviour of concrete reinforced with the modified fibre compared to concrete reinforced with non-modified PP fibre. C. Signorini et al. [130] have investigated the effect of silica-coated PP fibres on the mechanical properties of fibre-reinforced concrete. Polypropylene fibres were covered by silica nanoparticles using the sol–gel technique. They have found that nano-silica coating is an effective method to improve the bond strength in the fibre–cementitious matrix. The SEM analysis (Figure 12) shows that the surface of the fibre in a control sample appears scratched. Only in some places, mortar adheres to its surface, whereas, on the surface of modified fibres, attached mortar grains are visible.

### 4.3. Properties of Recycled Polymer Fibre-Reinforced Concrete

Recently, the possibilities of using recycled plastic waste fibres in concrete have attracted the attention of many researchers. Literature reviews indicate numerous experimental studies devoted to reinforcing concrete with recycled plastic fibres [131,132,133,134,135]. The attention is mainly focused on plastics found in wastes in a considerably large quantity. They include polyethylene terephthalate (PET) fibres, rubber aggregates and polystyrene wastes. The plastics are tested as a partial replacement for sand in concrete. An exchange ratio of 10% by volume could save 820 million tons of sand per year [134].

B.S. Al-Tulaian et al. [133] have investigated the effects of recycled PET waste fibres on the mechanical properties of Portland cement mortar, such as flexural strength, flexural toughness and shrinkage cracking. They found that the addition of fibres reinforced concrete, which was observed to display increased flexural toughness, as well as flexural strength. Moreover, increasing the fibre volume fraction leads to a significant improvement in minimizing plastic shrinkage cracking. All tested fibres differed in their length and volume fractions. Nevertheless, they caused a reduction in the total crack areas and crack widths.

Ochi et al. [134] examined the bond behaviour of concrete reinforced with recycled PET fibres derived from waste bottles. The samples with fibre content above 1% had a higher bending strength than the reference ones. A similar effect of enhanced concrete properties has been observed by Kim et al. [25], who studied the performance of concrete with different types of shredded, recycled PET. Embossed fibres showed superior mechanical bond strength, followed by crimped and straight fibres. Moreover, it was noticed that the samples with the highest bond strength also had the best resistance to plastic shrinkage cracking.

The influence of recycled PET fibres on the reinforced concrete’s early-age performance and mechanical characteristics has also been investigated. Borg et al. [31] used fibres shredded from waste plastic bottles of different sizes ranging between 30 and 50 mm and different fibre geometries (straight and deformed). They found that the addition of recycled PET fibres to concrete reduced the compressive strength regardless of the fibre profile. In contrast, the samples containing shorter fibres showed slightly better properties than the samples containing longer fibres. Furthermore, the addition of recycled PET fibres to concrete yielded restraints in crack development inducted by an environmental chamber. The most significant results were achieved in the case of the mixture containing the highest amount of 50 mm long deformed fibres, which is related to their better anchorage in the concrete matrix compared to straight fibres.

M. Horgnies et al. [136] studied the effect of PA wastes on the microstructure of lightweight mortars. In their study, sand was partially replaced by polyamide powder waste. The obtained results indicate that compressive strength was reduced proportionately to an increase in the content of polymer wastes. In contrast, the total porosity of lightweight concrete increased with the quantity of PA powder.

## 5. Conclusions

In summary, concrete strengthening by introducing polymer-based additives into the cement matrix has been studied. For this reason, the effects of the addition of superplasticizers, latexes and redispersible powders, admixtures, fibres and recycled polymers into concrete have been described. The examination of the literature allowed us to establish the following conclusions.

The addition of a plasticizer or superplasticizer allows the appropriate consistency to be achieved mainly by reducing the amount of water or the cement content.

The most important factors influencing the parameters of obtained concrete-based composites include the type, the number and the density of the adsorbing groups, length of the side-chain and its grafting density. Moreover, it should be stressed that the dosage and quantity of the superplasticizer used in the procedure has a significant impact on the adsorption efficiency of the superplasticizer onto cement particles. Redispersible powders and polymer dispersions affect the cement hydration process. In various forms, such as redispersible powders, latexes, liquid resins and water-soluble homo- or copolymers, they are able to form flexible polymer films after dehydration. Furthermore, they provide proper adhesion and cohesion in cementitious materials.

Polymeric fibres are known as materials characterized by elasticity, chemical resistance, high strength and excellent wear resistance. For this reason, cement-based materials containing fibres are characterized by improved mechanical properties, toughness, ductility and post-cracking resistance. In addition, it should be emphasized that the low melting temperature of polymeric fibres leads to the formation of concrete-based composites with reduced spalling at higher temperatures.

Based on the literature review, it was proved that polymer-based additives constitute valuable components of concrete that allow its limitations to be overcome. Prospectively, it is likely that further studies will focus on self-repairing concrete-based composites. Moreover, the most recent technological advances have been made in order to receive concrete without reinforcement, in the self-compacting technology, without scratches and cracks, with high aesthetic values and the highest quality. Most importantly, in the future, concrete will be lighter, safer, more flexible and durable, as well as environmentally friendly. The concrete of the future will also have the potential to use solar and wind energy, as well as capture and consume CO_2_ and NO_x_.

## Figures and Tables

**Table 1:** The most popular superplasticizers.

Name	Chemical Structure	Mechanism of Action	Ref.
Lignosulphonate (LS)	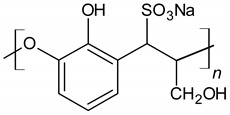	Electrostatic repulsion effect	[47,48]
Sulfonated naphthalene formaldehyde condensates (SNF)	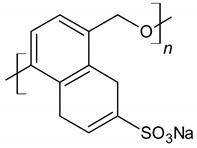	Electrostatic repulsion effect	[48,49]
Sulfonated melamineformaldehyde condensate (SMF)	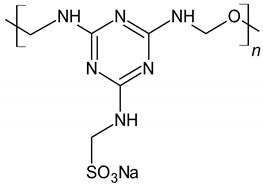	Electrostatic repulsion effect	[32,49,50]
Polycarboxylates (PCE)	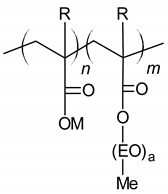 M = metal; R = Me, H; Me = methyl; EO = oxyethylene.	Steric hindrance	[51]

**Table 2:** The chemical structure of polymers.

Name	Chemical Structure	References
Styrene-butadiene rubber (SBR)	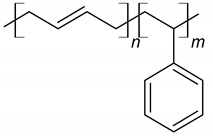	[88,89]
Polyacrylic ester (PAE)	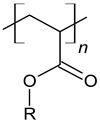	[12]
Poly (styrene-acrylic ester) (SAE)	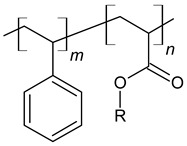	[6,12,92]
Poly (ethylene-vinyl acetate) (EVA)	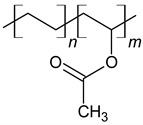	[14,83,84]
Vinyl acetate and versatate copolymer (VA/VeoVa)	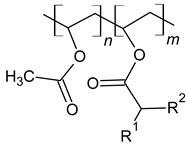 R^1^, R^2^ = alkyl groups	[15]

**Table 3:** Mechanical properties analysed in the case of cement–polymer materials.

Ref.	Type of Polymer	Curing Time	Dosage in Concrete	Compressive Strength	Flexural Strength	Tensile Strength	Toughness	Shrinkage	Bond Strength
[15]	VA/VeoVa powder	3 and 28 days	Powder-to-cement ratio by mass of 0–20%	+	+	-	+	+	-
[6]	SA	3 and 28 days	0, 3, 5, 10 and 15 wt%	+	+	-	-	-	+
[12]	SBR. SAE. PAE	1, 7 and 28 days	0, 10 and 20%	+	+	-	-	+	-
[14]	EVA and acrylate copolymer	(1) 28 days in humid chamber, 14 days in dry chamber. (2) 1 day in humid chamber, 41 days in dry chamber	0 and 10%	+	-	+	-	-	+
[18]	Redispersible acrylic polymer powder	From 2 h to 90 days	0, 2, 6 and 10%	+	-	-	-	-	-
[89]	SBR	7, 28 and 56 days	0, 5, 10, 15, 20 and 25%	+	+	-	-	-	-
[92]	EVA, SBR and SAE	28 days	Polymer–cement ratios (P/C) 0, 5, 10, 15 and 20%	-	-	-	-	-	-
[98]	Siloxane-based RPP	1 h; 1, 7 and 28 days	0.117, 0.233, 0.350, 0.467, 0.583 and 0.700%	+	+	+	-	-	+
[99]	Redispersible latex powder	3 days	0–4%	+	+	-	-	-	-

+: studied parameters, -: omitted parameters.

**Table 4:** Basic characteristics of fibres.

Fibre	Sp. Gravity (kg/m^3^)	Tensile Strength (MPa)	Modulus of Elasticity (GPa)	Elongation at Break (%)	References
Polypropylene (PP)	0.90–0.91	325–770	3.5–4.2	15–20	[21,23,26,28,106]
Polyethylene (PE)	0.97	2610	79	4–100	[4,107]
Polyamide (PA)	1.14	900–970	3.5–6.8	16–21	[108]
Polyvinyl alcohol (PVA)	1.26–1.30	1529–1600	45	6–7	[107,109]
Steel (ST)	7.80	400–2500	200	3.5–18	[21,26,106]
Cellulose	1.20	300–500	10	-	[107]

**Table 5:** Mechanical properties of cementitious materials containing fibres.

Ref.	Type of Polymer Fibres	Dosage in Concrete	Length (mm)	Shape	Specific Gravity	Tensile Strength (MPa)	Elasticity Modulus (GPa)
[116]	High-performance PP fibres	0.4, 0.6 and 0.8% by volume	48	Continuously embossed	0.90–0.92	550	10
[117]	PP and PVA	1%	12 (PP), 8 (PVA)	Circular and smooth (PP), circular and rough (PVA)	0.95 (PP),1.3 (PVA)	400–550 (PP), 1600 (PVA)	5.6 (PP), 42 (PVA)
[118]	PP	0, 3, 6, 9 and 12 kg/m^3^ for cement content	45	Wavy shape	0.91	320	5.88
[119]	PP	Mixes of staple fibres at 0.6 kg/m^3^ with coarse synthetic monofilament fibres at 3, 6 and 9 kg/m^3^ to concrete	60, 10–25	Coarse monofilament and staple fibres	-	320550	5.88 4.2
[28]	PP	0.7, 0.9, 1.5, 2 and 4 kg/m^3^	12	Monofilament	0.91	400	3.5–3.9
[106]	PP	0.5% and 1.0%	12	Straight	0.91	400	-
[112]	PP Nylon	Volume fractions of 0.25, 0.50 and 0.75%	12 19	Fibrillated and multi-filament	0.90 1.13	300 400	-
[113]	PVA	0.1%	6	-	1.26	1600	45
[114]	PE	1.0, 1.5, 2.0 and 2.5%	12	-	0.97	19502700	3982
[110]	PP	1.0%	15	Straight, round	0.9	800	8
[108]	PA	0.25, 0.5 and 0.75%	12 54	-	1.14	900 970	3.5–6.8 5.15
[109]	PVA	0, 0.2, 0.5, 0.8 and 1.0%	6	Smooth and straight	1.30	1529.5	-

## Data Availability

The data presented in this study are available on request from the corresponding author.
